# Testing the ego-depletion effect in optimized conditions

**DOI:** 10.1371/journal.pone.0213026

**Published:** 2019-03-07

**Authors:** Rémi Radel, Mathieu Gruet, Krystian Barzykowski

**Affiliations:** 1 LAMHESS, Université Côte d’Azur, Nice, France; 2 LAMHESS, Université de Toulon, Toulon, France; 3 Applied Memory Research Laboratory, Jagiellonian University, Kraków, Poland; Universidad Autonoma de Madrid, SPAIN

## Abstract

The observation that exerting self-control in an initial task impairs subsequent self-control performance in a following task has been used to explain a wide range of phenomena. If evidence for this “ego-depletion” effect was initially believed to be strong, it is now questioned. Recent meta-analyses indicated that this effect was sensitive to publication bias and that it was greatly reduced after control for this bias. In a pre-registered replication attempt where an ego-depletion protocol was conducted in multiple independent laboratories, the effect was not distinguishable from zero. Here, a different approach is adopted to examine the validity of this effect by improving the experimental protocol with the addition of important methodological precautions: 1) a pre-test measurement, 2) a learning period, 3) a prolonged depleting task, 4) a similar control condition, and 5) valid indexes of self-control. Accordingly, a well-learned Simon task was done before and after 1h of continuous practice of a Stroop task in a high inhibition demands condition (75% of incongruent trials) or in a control condition (0% of incongruent trials). Datasets from between-subjects (Study 1, *N* = 82) and within-subjects (Study 2, *N* = 52) experiments were analyzed using generalized linear mixed models. A significant ego-depletion effect was found in Study 1 (greater interference effect and accuracy decline in high inhibition demands than in control condition) but not in Study 2. Because it is difficult to explain this difference in results, the findings suggest that, even in a context chosen to optimize the observation of an ego-depletion effect, it seems difficult to be conclusive about the existence of this effect.

## Introduction

Self-control, commonly defined as the capacity to actively override impulses, to suppress urges, and to resist habits and temptations [1], is crucial for a large variety of activities and to achieve long-term goals and plans. However, it has been suggested that self-control is a finite resource that can be easily depleted [1]. This « ego-depletion » perspective was formulated following the observation that exerting self-control in a first task led to an impaired ability to exert self-control in a second task [2]. An impressive number of studies has then followed in this vein, testing a similar two sequential tasks protocol in a variety of contexts with the aim of explaining the occurrence of some maladaptive behaviors as a result of a self-control failure. Despite this large set of studies, it is however still impossible to be conclusive on the existence of an ego-depletion effect, and it has therefore been recommended to conduct additional research to indicate if this effect really exists [3,4].

While a first meta-analysis including 198 studies established evidence for this effect with a moderate to large average effect size (Cohen’s *d* = .62) [5], this result was later challenged [6,7,8,9]. For example, a subsequent meta-analysis showed a publication bias in the corpus of studies and indicated that effect size was no longer different from zero after accounting for this bias [7]. A more recent meta-analysis with adequate control of the publication bias and a more careful selection of the studies, including only the studies with tasks directly associated with self-control, still indicated a significant effect size, but of a smaller amplitude than in the original meta-analysis (Hedge’s *g* = .38 and .24 after bias correction) [8].

To overcome this lack of homogeneity in the results from these meta-analyses, another approach towards examining the evidence for ego-depletion has been to conduct a large standardized pre-registered replication project in multiple independent laboratories across the world [9]. The protocol chosen for replication was taken from Sripada et al. [10]. The first task lasted 7min 30s and required participants to either press a button when a word contained the letter “e” (control condition) or do the same but withhold the response if the “e” was next to or one letter away from a vowel (depletion condition). The subsequent task was a multisource interference task combining spatial and flanker interference when giving an answer regarding the identity of a target number in a sequence of three. The practice for this task consisted of 20 practice trials. Overall results obtained in 23 laboratories with a total of 2141 participants indicated a trivial effect size not distinguishable from zero. While this approach has some merits by providing a valid estimation of the effect size with high statistical power and no publication bias, it has also been criticized as reflecting only a very specific context that is not really appropriate for observing the ego-depletion effect [3,11]. Specifically, it has been suggested that the depleting task may not be effective as it may actually require only minimal self-control resources [11], may not be long enough [12], and may depend upon individual differences [13].

### The present research

In order to gather more evidence concerning the existence of the ego-depletion effect, we adopted yet another approach for examining this question by trying to improve the methodological standards of the two sequential tasks protocol [3,4]. In other words, we have tried to maximize the conditions for the observation of the ego-depletion effect to make sure that this effect truly exists. If a reliable effect is found, it could help to end the debate and provide a valid protocol to examine the consequences of self-regulation failure. To achieve this goal, we selected tasks that are known to mobilize self-control resources, chose a task duration that has more chances to induce performance decrement, and used precautions to minimize individual differences. Each of these factors are discussed below.

First, a large variety of self-control tasks has been used in the previous studies (e.g., a handgrip task, eating healthy unpleasant food, controlling one’s own emotions when watching a movie, balancing on one leg) which has led to a very large and loose definition of self-control. As noted by Lurquin & Miyake [14], many tasks were not chosen because they primarily tap self-control resources but because they were used before and had previously shown a depleting effect or because they were simply demanding. However, the theoretical models of self-control indicate that self-control is supposed to primarily rely on inhibitory control [1,15] by blocking automatic responses and impulses. Therefore, we believe that the selected tasks should be reliably associated with inhibitory control. In our study, we chose conflict tasks that include incongruent trials where an automatic response has to be inhibited to comply with the instructions of the task. It has been well-demonstrated that such tasks mobilize the cerebral structures that have been associated with cognitive inhibition such as the inferior frontal cortex, dorso-lateral prefrontal cortex, anterior cingulate cortex, or supplementary motor area [16]. Another advantage of these tasks is the ease with which it is possible to manipulate the degree of inhibition demands by manipulating the percentage of incongruent trials in the task. Using this possibility, we created a very similar control condition where participants did the same task but without incongruent trials. Since it has been previously shown that transfer effects from one task to another are particularly visible when the two tasks rely on the same cognitive processes [17], the existence of several conflict tasks also allowed us to choose two tasks relying on similar neural processes. Specifically, a Simon task was used as a diagnostic of self-control performance as this task provides a small but reliable interference effect (i.e., the difference between incongruent and congruent trials response time, [18]) and the Stroop task was used as the depleting task as it led to higher inhibition demands by leading to an important interference effect.

Second, the duration of the depleting task may also be an important parameter to consider. Meta-analyses showed that longer duration led to a higher effect size [5,8]. An experimental study manipulating the task duration also found that the ego-depletion effect was only present in longer durations [19]. It should be noted that the large majority of studies have used very short task duration below 10 minutes [5]. For such short duration, it actually seems unlikely that any effects would be observed, as time-on-task studies that observe the evolution of performance over time have rarely reported decrements in performance in inhibitory control tasks earlier than 40 min of continuous practice [20,21]. We believe that if ego-depletion really reflects an inability to maintain an optimal performance, it should then correspond with time-on-task effect. For this reason, we chose a long continuous practice of 60min of the Stroop task that should give enough time for time-on-task decline.

Third, another problem identified in previous ego-depletion studies is the large variability in self-control performance that could explain the heterogeneity of the results. First, it was shown that large differences can exist in self-regulation performance and that these differences can explain the presence of the ego-depletion effect [13]. For this reason, we used a pre-test measurement on the Simon task to really track the evolution of this performance as a consequence of the depleting task. In this manner, the differences seen in the post-test cannot be explained simply by group differences and a possibly unsuccessful randomization. Because variability is important in the early practice of a task but reduced after training [22], we also trained participants on the Simon task until they showed stable performance on the task in order to reduce the variability in performance. Finally, in addition to the standard between-subject design, we also examined data from a within-subject design that better controls for individual differences.

**Fig 1:**
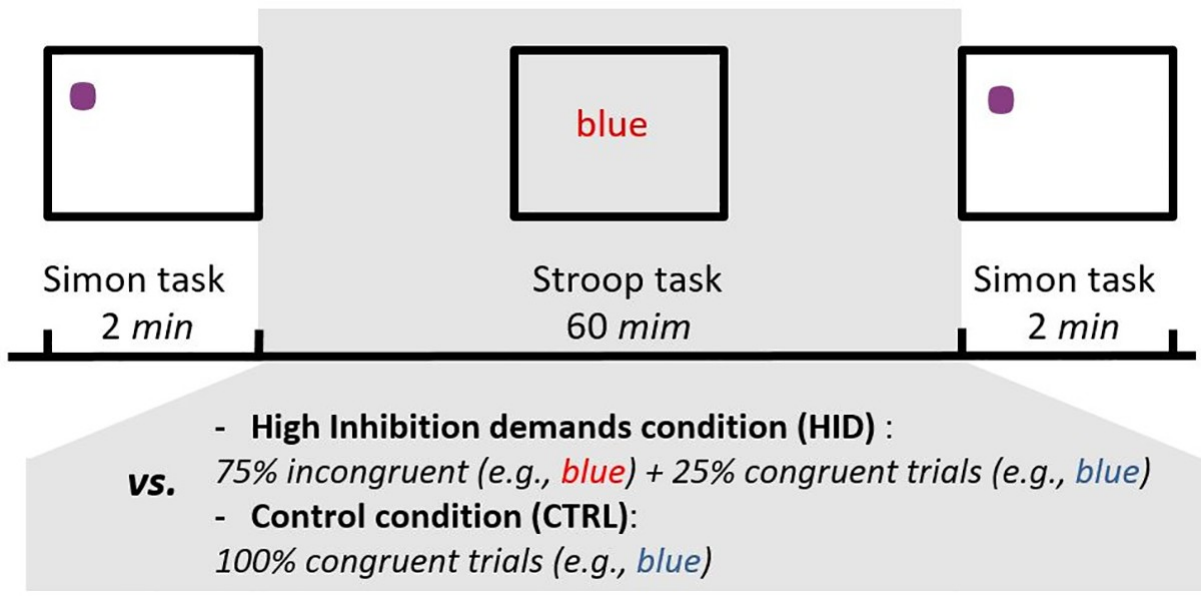
Illustration of the protocol used in Study 1 and Study 2. Note that Study 1 used a between-subjects design to manipulate the inhibition demands required in the Stroop task whereas Study 2 used a within-subjects design to manipulate inhibition demands.

In summary, we tested an improved version of the two sequential tasks protocol that was optimized for the observation of the ego-depletion effect (see Fig 1 for an illustration of the protocol). It is therefore expected that this protocol should lead to an ego-depletion effect (i.e., increase in the interference effect after the continuous practice of the Stroop task with high inhibition demands). Two datasets were analyzed, one coming from a between-subjects study (Study 1, *N* = 82) and another one coming from a within-subjects study (Study 2, *N* = 52). A sensitivity power analysis conducted on each study indicated that small effect sizes (Cohen’s *f* = .10) could be detected in both studies with an alpha level of .05, a power level of .80, and a correlation among repeated measures of .80 (estimated from a previous study conducted in our lab with the same Simon task also used as a repeated measure, [23]. The sensitivity power analysis was conducted with GPower [24] by indicating ANOVA (“repeated measures: within-between interaction” for Study 1 and “repeated measures: within factors” for Study 2) as statistical tests. However, the real statistical power is certainly even slightly higher as we actually used generalized linear mixed model (GLMM), which was found to be more powerful than repeated ANOVA measures [25]. It should also be noted that these datasets come from a larger experimental program on the role of cognitive inhibition in involuntary mental time travel. However, only behavioral performance in the conflict tasks are reported and analyzed here while the whole study analyzing the involuntary mental contents is reported elsewhere [26]. These additional measures were collected after the post-test Simon task. All measures and manipulations until the post-test Simon task are reported in this manuscript. Data are available online on Mendeley Data [27].

## Study 1

### Participants and design

The design was a randomized between-subjects design. A total of 82 Polish students participated, and were paid 50 PLN (equivalent to 14$ USD). The two groups were matched in size and in the distribution of sex and age. Accordingly, the high inhibition demands (HID) group was composed of 41 participants (27 females, *M*_*age*_ = 22.34, *SD* = 1.67, range 19–25 years) and the control (CTRL) group also included 41 participants (28 females, *M*_*age*_ = 22.39, *SD* = 1.89, range 19–28 years). This study was approved by the Research Ethics Committee of the Institute of Psychology at Jagiellonian University. Written consent for participation was obtained prior to data collection.

### Materials

#### Manipulation of inhibitory control: The Stroop task

Two versions of a Stroop task [28]] were used to manipulate the level of inhibition required. The task consisted of four types of stimuli, which were Polish translations of the words red, green, blue, and yellow. Each word was displayed on a computer screen in any of these four ink colors. Participants were instructed to react as fast as possible in response to the color of the ink of the word, without paying attention to the meaning of the word. They answered by pressing the key (‘R’ for blue, ‘F’ for green, ‘J’ for red and ‘I’ for yellow) with the sticker corresponding to the color of the ink. Each word was displayed for 2000 ms with 150 ms interval between trials and a 1000 ms feedback screen after each trial. While the meaning of the word and the color of the ink were the same in congruent trials, the meaning differed from the color of the ink in incongruent trials. In the high inhibition demands version, 75% of trials were incongruent and 25% of trials were congruent. In the control version, 100% of trials were congruent. Both of these tasks lasted 60 minutes and were presented on a computer using E-prime 2.0 (PST, Pittsburgh, USA). Finally, for learning purposes we also used a short 2-minute version of the Stroop task that consisted of 50% of congruent and incongruent trials. The mean reaction time and percent of correct responses were automatically calculated at the end of this learning task.

#### Diagnostic of self-control performance: The Simon task

The Simon task consisted of a yellow or pink circle appearing either on the left or on the right side of the screen. Participants were instructed to respond as fast as possible by pressing a left (‘S’) or right (‘L’) key of the keyboard with the left or right index finger according to the color of the stimulus (left-pink; right-yellow). The stimuli appeared after a fixation cross (600ms) at the center of the screen. In congruent trials, the spatial location of the stimulus and response were the same (e.g., right stimulus/right response). In the incongruent trials, the spatial location of the stimulus was opposed to the location of the response (e.g., right stimulus/left response), which triggered an automatic response that had to be inhibited. The Simon task consisted of 160 trials, which lasted about 2 minutes. At the completion of the task, participants mean RT (RT) and percent of correct responses were presented on the screen.

### Procedure

In a first part of the session, participants were initially familiarized with each task. They started the training with the Simon task. Each participant did the task at least four times. If participant accuracy and RT on the fourth task varied less than 5% with the last task, the training was stopped, otherwise the task was completed again until these criteria were met. To be retained in the study, participant responses also had to be sufficiently fast (below 700 ms) and accurate (above 80% of correct responses). The performance in the last task was considered as the baseline performance (pre-test). Then, participants were familiarized with the Stroop task by performing the 2-min version of the Stroop task. Then, if no anomalies were found in their responses, participants started the continuous practice of the Stroop task for 60 minutes either with a low or high inhibition demands depending on the conditions (CTRL vs. HID). Right after the completion of the Stroop task participants did the Simon task again (post-test) in order to estimate the alteration of the inhibition performance induced by the prolonged practice of the Stroop task.

### Data analysis

First, it should be noted that data analysis was performed only at the end of the planned data collection period to avoid any temptation to stop data collection after looking at the results, a practice that can artificially create an effect [29]]. In addition, the statistician was kept blind to the experimental condition using a coding scheme. No participant was excluded from the analysis.

To evaluate the ego-depletion hypothesis, performance of the pre- and post-test Simon tasks were analyzed. The dependent variables that were extracted from the Simon task were RT and accuracy. The interference effect, computed from RT data constituted the primary outcome of the study (see below).

#### Response time

The first trial of each task was disregarded as they quite often led to abnormally long RT due to the adjustments made by the participants in responding to the task. Only correct responses were kept for the analysis. Short responses (below 100ms) were also excluded as they reflected anticipation errors. Rather than averaging trials into blocks, which results in a loss of information [27], the distribution of all trials was analyzed using a mixed model approach. As noted in methodological studies of the analysis of RT [30,31,32], distributions of RT are positively skewed because they rise rapidly on the left and have a long positive tail on the right. To accommodate the skewed distribution of RT, a GLMM modeled for gamma distribution (with an identity link) was used [33]]. A random intercept effect structured by subjects was included to control for the non-independence of the data. The fixed factors of the GLMM were the time of measurement (pre-test *vs*. post-test), the type of trials (congruent *vs*. incongruent trials), the condition (CTRL *vs*. HID), and all interactions between these factors.

#### Interference effect

The interference effect corresponds to the time needed to resolve the interference created by the activation of the inappropriate automatic response tendency in incongruent trials (i.e. the spatial location of the stimulus in the Simon task). The interference effect is therefore calculated as the difference between the mean RT of incongruent trials and the mean RT of congruent trials. This index is therefore considered as a good indicator of the inhibition performance with a greater value representing more difficulty to exert the inhibitory process. As these values are usually normally distributed [23,34] linear mixed models (LMM) were used for this variable. A random intercept effect structured by subjects was included to control for the non-independence of the data. The fixed factors of the LMM were the time of measurement (pre-test *vs*. post-test), the condition (CTRL vs. HID), and the interaction between these two factors.

#### Accuracy

The first trial of each block, anticipation errors, and omissions (no-response) were removed from the analysis. Since the dependent variable is a dichotomous variable (0 = errors; 1 = correct responses), it can be easily modeled using a GLMM for a logistic distribution [35]]. Apart from the fitted distribution, the specification of the model was the same as on RT.

To report descriptive statistics, we provided the means ± standard errors estimated by the models in order to actually represent the effect of interest after controlling for other factors and covariates.

### Results

#### Response time

**Fig 2:**
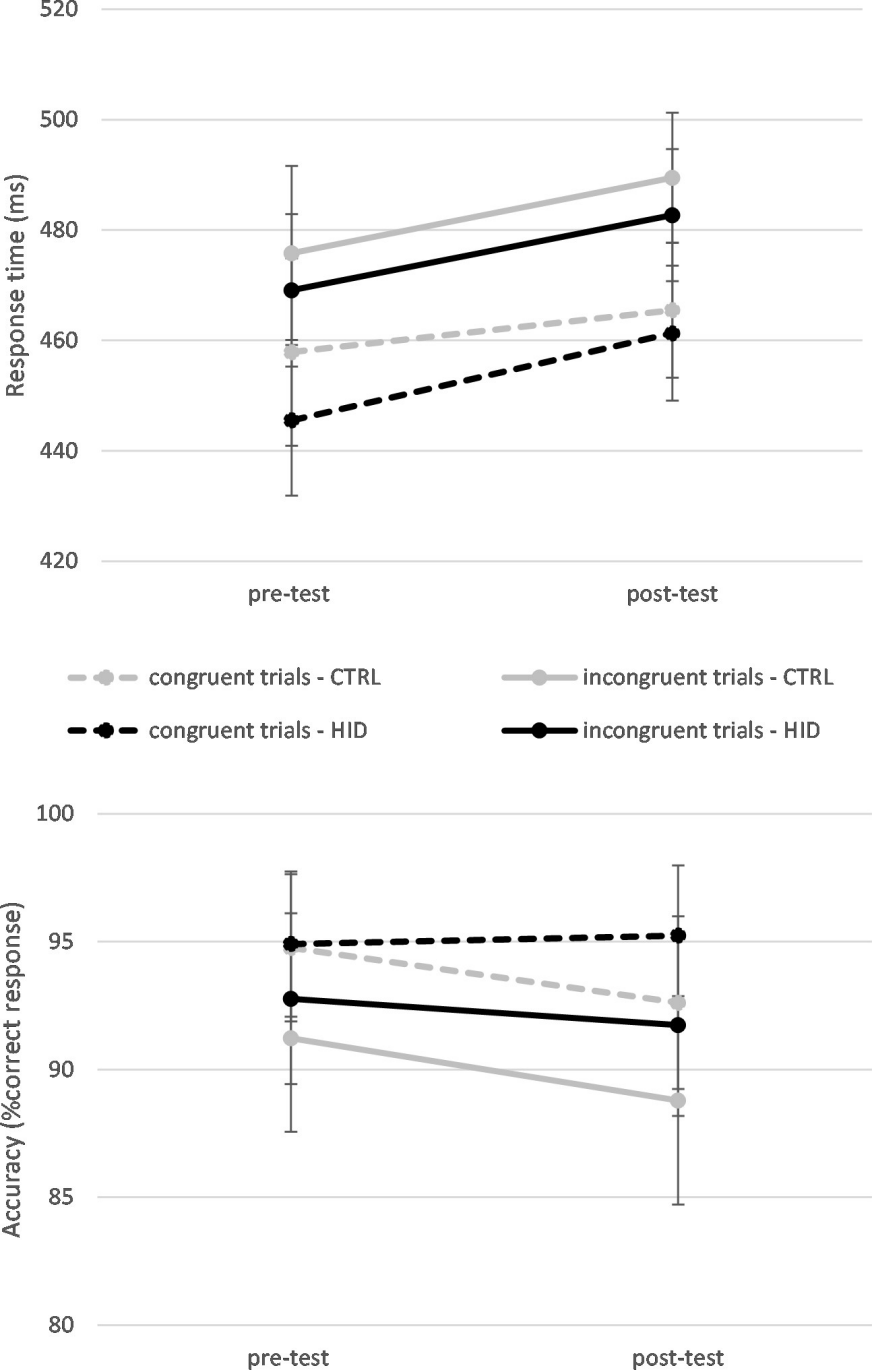
Descriptive statistics (exact means and standard errors of the mean) representing the evolution of response time (upper panel) and accuracy (lower panel) as a function of the time of measurement (pre-test *vs*. post-test) and as a function of the level of inhibition in the Stroop task (control condition, CTRL *vs*. high inhibition demands, HID) in Study 1.

A total of 8.88% of the data was excluded, because it corresponded to errors (7.20%of the data) or to anticipation and omissions (0.68% of the data). Descriptive statistics are illustrated in Fig 2. The GLMM indicated a significant effect of the time of measurement with slower RT in the Simon task done after (471±5ms) than in the Simon task done before the Stroop task (459±5ms), *F*(1, 24163) = 71.803, *p* < .001. A congruence effect was also found with slower RT in incongruent (476±5ms) than congruent trials (459±5ms), *F*(1, 24163) = 227.919, *p* < .001.

#### Interference effect

**Fig 3:**
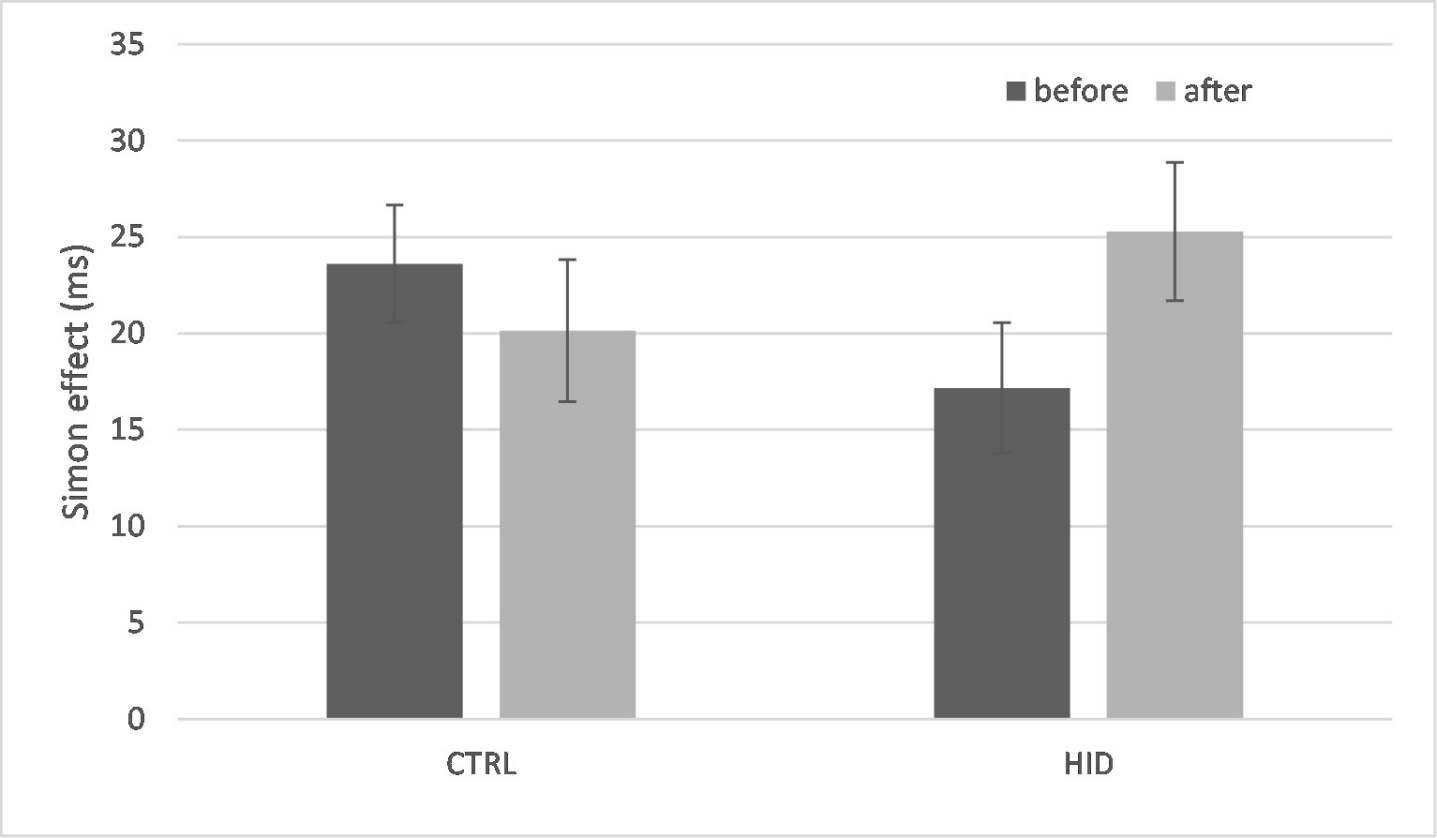
Descriptive statistics (exact means and standard errors of the mean) representing the evolution of the interference effect (the difference between response time of incongruent trials and response time of congruent trials) as a function of the time of measurement (pre-test *vs*. post-test) and as a function of the level of inhibition in the Stroop task (control condition, CTRL *vs*. high inhibition demands, HID) in Study 1.

Descriptive statistics are reported in Fig 3. The LMM indicated a significant time by condition effect, *F*(1, 80) = 4.850, *p* = .031. While the interference increased from the pre-test (17.2±3.4ms) to the post-test (25.3±3.4ms) for the participants who made the Stroop task with high inhibition demands (*p* = .044), the interference did not significantly change from the pre-test (23.6±3.4ms) to the post-test (20.1±3.4ms) for the control participants.

#### Accuracy

A total 0.68% of the data was excluded as it corresponded to anticipation errors or omissions. Descriptive statistics are illustrated in Fig 2. The GLMM indicated a significant effect of the time of measurement with a lower accuracy in the Simon task done after (93.5±0.5% correct responses) than in the Simon task done before the Stroop task (94.6±0.5% correct responses), *F*(1, 26051) = 15.099, *p* < .001. A congruence effect was found with less accuracy RT in incongruent (92.5±0.6% correct responses) than in congruent trials (94.8±0.4% correct responses), *F*(1, 26051) = 101.881, *p* < .001. A time by condition interaction was also found, *F*(1, 26051) = 7.107, *p* = .008. While participants who made the Stroop task with the high inhibition demands were less accurate after (91.9±0.6% correct responses) than before the Stroop task (94.0±0.6% correct responses) (*p* < .001), the accuracy of participants in the CTRL condition did not change from the pre-test (95.1±0.4% correct responses) to the post-test (94.8±0.4% correct responses). However, no time by condition by trial type interaction was found.

### Discussion

Because high level of inhibition in the Stroop task led to reduced accuracy, the different evolution of the time needed to perform inhibition found between the groups cannot be explained in terms of a possible change in the speed-accuracy trade-off. In sum, this suggests that the manipulation of the level of inhibition required in the Stroop task was really effective to affect self-control performance, leading to the expected ego-depletion effect.

## Study 2

### Participants and design

The design was a within-subjects design. The level of inhibition demands (high inhibition demands, HID vs. low inhibition demands, CTRL) were manipulated by the completion of two sessions, each one on separate days (average distance = 7.11 ±.73 days, range = 6 to 10 days). To keep the experimental sessions as comparable as possible in terms of the time of day and activities, the second session was scheduled one week later, at the same time and day as the previous one every time it was possible. The order of the conditions was randomized and counterbalanced among participants. A total of 52 participants (27 females, *M*_*age*_ = 23.12, *SD* = 2.33, range 19–31 years) were recruited. They received 100 PLN (c.a. 28$ USD) after the study completion. It should be noted that 10 participants completed only one session, but their partial data were still analyzed, so that no participant was excluded from the analysis. This study was approved by the Research Ethics Committee of the Institute of Psychology at Jagiellonian University. All participants signed an informed consent form before taking part to the study.

### Procedure

The experimental sessions were exactly similar to those of Study 1. It should be noted that participants also did the training when they came for the second session and they completed the same amount of training blocks as in their first session to keep the two sessions identical.

### Data analysis

The same data analysis strategy was used in Study 2. However, it should be noted that a session factor and its interactions with the other factors were added in the GLMM and LMM models to control for the order of the sessions.

### Results and discussion

#### Response time

**Fig 4:**
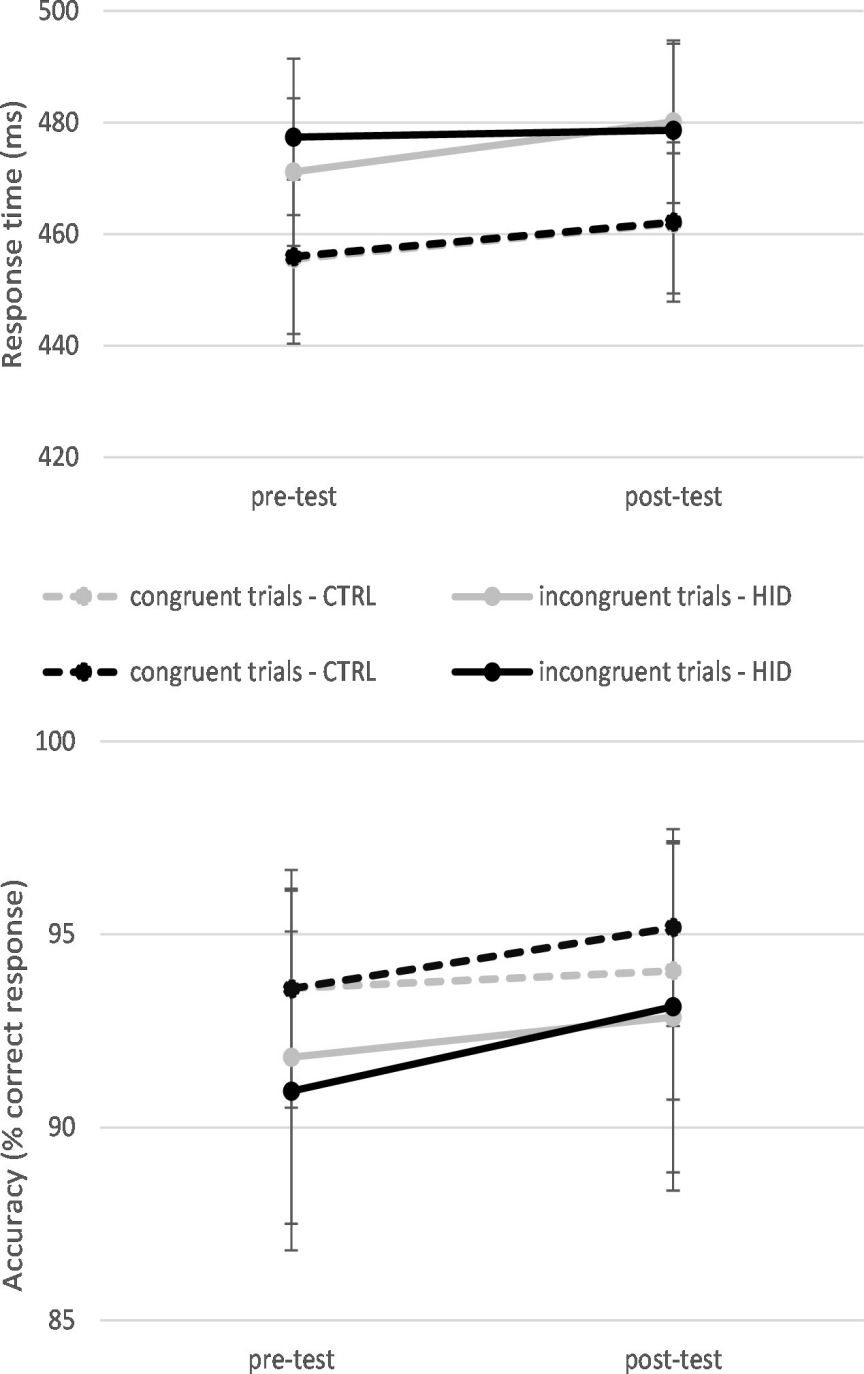
Descriptive statistics (exact mean and standard error of the mean) representing the evolution of response time (upper panel) and accuracy (lower panel) as a function of the time of measurement (pre-test *vs*. post-test) and as a function of the level of inhibition in the Stroop task (control condition, CTRL *vs*. high inhibition demands, HID) in Study 2. Note that the data of response time for the congruent trials in the control condition is not visible on the graph as it is located exactly under the data of response time for the congruent trials in the high inhibition demands condition.

A total of 7.26% of the data was excluded, because it corresponded to errors (6.78%of the data) or to anticipation errors (0.48% of the data). The GLMM indicated a significant effect of the order of the session with quicker RT in the Simon tasks done during the second session (455±6ms) as opposed to the first (479±6ms), *F*(1, 29927) = 401.966, *p* < .001. A significant effect of the time of measurement was found with slower RT in the Simon task done after the Stroop task (470±6ms) rather than in the Simon task done before (463±6ms), *F*(1, 29927) = 33.812, *p* < .001. A congruence effect was also found with slower RT in incongruent trials (476±6ms) rather than congruent ones (458±6ms), *F*(1, 29927) = 223.530, *p* < .001. The interaction between the order of the session and the time of measurement was significant, *F*(1, 29927) = 20.134, *p* < .001. Specifically, while the first session led to a significant slow-down of RT from the pre-test (473±6ms) to the post-test (485±6ms) (*p* < .001), this effect was no longer visible in the second session (from 454±6ms to the pre-test to 455±6ms in the post-test). Finally, a time by condition effect was found, *F*(1, 29927) = 4.498, *p* = .034. The increase of RT due to the continuous practice of the Stroop task was more important in CTRL (from 462±6ms to the pre-test to 472±6ms in the post-test) than HID (from 464±6ms to the pre-test to 468±6ms in the post-test). Fig 4 represents the mean RT as a function of the condition, the time and congruence.

#### Interference effect

The LMM indicated a significant session effect, *F*(1, 141) = 11.058, *p* = .001, suggesting a smaller interference in the second (13.7±2.0ms) than in the first session (22.0±2.0ms). The session also significantly interacted with the time of measurement, *F*(1, 141) = 4.010, *p* = .047. While the interference increased numerically but not significantly from the pre-test (17.2±2.8ms) to the post-test (25.3±2.8ms) in the first session (*p* = .044), the interference decreased numerically but not significantly from the pre-test (23.6±2.8ms) to the post-test (20.1±2.8ms) in the second session. No main or interaction effect of the condition was found (*p*s>.05). It should however be noted that numerical values go in the opposite direction to what could be expected from the ego-depletion effect, with a decrease of the interference from the pre-test (21.2±2.8ms) to the post-test (16.1±2.8ms) in the HID condition.

#### Accuracy

A total 0.48% of the data was excluded as it corresponded to anticipation errors or omissions. The GLMM indicated a significant effect of the order of the session with a lower accuracy in the second (93,8% correct responses) than in the first session (94.8% correct responses), *F*(1, 32111) = 15.903, *p* < .001. The GLMM indicated a significant effect of the time of measurement with a better accuracy in the Simon task done after (95.0% correct responses) than in the Simon task done before the Stroop task (93.7% correct responses), *F*(1, 32111) = 25.869, *p* < .001. A congruence effect was also found with less accuracy RT in incongruent (93.5% correct responses) than in congruent trials (95.0% correct responses), *F*(1, 32111) = 34.606, *p* < .001. No main of the condition (HID vs. CTRL) and no two or three levels interactions of the condition with the type of trials or/and the time of measurement were found (*p*s>.05).

### Discussion

In this within-subject research, we found no effect of the depleting manipulation on interference time and accuracy. While a condition effect was found in the general RT, this effect was rather in the opposite direction of what could be expected from an ego-depletion hypothesis. In sum, this study provided no evidence for the ego-depletion effect.

## General discussion

The present research aimed at testing the existence of the ego-depletion effect using another approach by testing the presence of this effect in a context where we optimized the experimental conditions for the observation of this effect. To achieve this goal, we selected tasks that are known to mobilize self-control resources, chose a task duration that is long enough, and used precautions to minimize individual differences. We analyzed data from two different studies, a first study with a between-subjects design and a second study from a within-subjects design. The results were contrasted with different patterns of results in the two studies.

In Study 1, RT drops in both the CTRL and HID conditions but a significant interaction effect could be seen on the interference effect as only the participants HID showed an increase of the interference time from the pre- to post-tests. In addition, accuracy also only declined significantly from pre- to post-test for the participants in high but not for those in the CTRL inhibition condition. In sum, these results clearly indicate the presence of an ego-depletion effect, as the inhibition performance was reduced after prolonged mobilization of this resource in the preceding Stroop task. Nevertheless, it should be noted that the observed effect is still relatively small given the precautions that have been taken to maximize the effect. It is expected that such an effect would have limited practical implications in real life.

This finding was not replicated in Study 2 when employing a within-subjects design. Specifically, RT slowed down more from pre- to post-test in the CTRL than in the HID condition. No other condition effect was seen on interference and accuracy. However, strong effects of the session order were found on RT, interference, and accuracy, which could have masked the effect of ego-depletion. For example, an increase of the interference effect from the pre- to the post-test was only visible in the first but not in the second session. Although we ran our participants through a long learning procedure to ensure a stabilized performance, it seems that participant performance still evolved with additional exposure to the task. Even if the task order was counterbalanced and statistically controlled, the importance of this effect certainly interfered with the effect of interest. As such, it is possible that the within-subject design is not adapted to examine the ego-depletion effect due to the difficulty to control for learning effect. If an even longer learning protocol could ensure a more stable performance, it is possible that participant response strategies would become so refined that incongruent trials would then require only a minimal amount of inhibition. Nevertheless, it should still be noted that we did not observe an interaction between the condition and the session on our main dependent variable, i.e., the interference effect, which suggests that there was no ego-depletion pattern on any of the sessions. For example, if only the first session was considered, it would thus bring us back to the exact same between-subject design as in Study 1 due to the counterbalancing of the conditions. However, the results do not correspond to those of Study 1. The absence of an ego-depletion effect in Study 2 can certainly not be explained by an inadequate level of statistical power as we used a relatively large sample size in a within-subject design and used statistical analyses that optimize the statistical power [25]. In addition, it is not even possible to assume that the effect was too small to be detected, because the numerical values indicated that the effect was rather in the opposite direction of what would be expected from the ego-depletion effect.

In sum, our findings confirm the lack of consistency of the ego-depletion effect [6,9]. Even with an experimental paradigm that was optimized for the occurrence of this effect (long duration of the task serving to manipulate the inhibition resources, two similar conflict tasks to facilitate transfer effects), we were not able to observe this effect in a systematic manner. While this study was not pre-registered, it would be difficult to argue that this result is subject to a publication bias as reporting inconsistent results rather leads to a reduction of the chances of being published [36]].

### Limits

A first limit could be the duration of our depleting task. Our depleting task was uncommonly long in comparison to traditional ego-depletion studies. In previous ego-depletion studies, much shorter tasks were typically used with a duration between 4 and 10 minutes [5]. This large difference in task duration between our studies and the previous studies can make the comparison of results more difficult because long tasks can imply other mechanisms (task monotony, boredom, or habituation). However, because ego-depletion was initially explained as a fatigue effect [1], we believed that it was important to have a depleting task that is long enough to truly lead to resources depletion. In time-on-task studies, very long periods of continuous practice tasks are typically used, often as long as 3 hours of continuous practice [37,38,39,40], and these studies have shown that inhibitory control performance rarely decreased before 40 min [20,21,37]]. An interesting study also showed that even after 2 hours of continuous practice of a conflict task, an unexpected monetary reward provided to the participants for their performance in a supplementary block led them to to perform as well as in the early stages of the task [40]]. Such result indicates that self-control capacity can be in fact quite robust to fatigue. In line with the recent theoretical reconsideration of the ego-depletion effect [41]], it is possible that motivational factors are certainly more important to explain variations in self-control performance. In this regard, the lack of control over these motivational factors may represent a limit of our study. While we have striven to design an ego-depletion protocol with high methodological standards, we did not control how much participants were involved in the depleting task. We therefore recommend including reward-based performance in the depleting task to increase participant motivation to perform at their best [42].

### Perspectives

It is certainly difficult to suggest such a recommendation while so much effort has already been made to study this effect, but in line with other authors [3,4], we believe that more research is still needed before pronouncing a judgment concerning the existence of the ego-depletion effect. Nevertheless, to optimize this effort, we also believe that the orientation of future research should be guided by a greater consideration of the literature on cognitive fatigue, and more particularly on the different time-on-task effects for each cognitive function. As noted earlier [14], the cause of the problem certainly lies in the lack of a clear operational definition of self-control. Because self-control has been said to rely primarily on inhibitory control [15,43][], this is the function we focused on in this research. Nevertheless, inhibitory control is more robust than other functions related to fatigue effects [44][], and more decrements could certainly be seen by considering other cognitive functions. For example, very quick and reliable decrements can be seen on vigilance and sustained attention [45][] or working memory [46][]. So, it is possible that an ego-depletion effect could be visible when considering other cognitive functions. For this reason, we first call for conceptual work and for a consensual definition of self-control, detailing more precisely the situations that requires self-control and the cognitive functions that are mobilized in these situations, and then it will be possible to examine the existence of the ego-depletion effect in a protocol that specifically targets these cognitive functions.

## Conclusion

Taken together, our results suggest that when manipulating the level of inhibition demands during the practice of long and continuous task, we could not systematically observe an ego-depletion effect. In other words, even in a protocol that was designed to optimize the conditions of its occurrence, ego-depletion remains an elusive effect. For the future, we see value in additional research on ego-depletion but first recommend conceptual work to better define the cognitive attributes of self-control. For the moment, this study indicates that if self-control just relies on inhibitory control, then it might be hard to observe ego-depletion.
